# Correction to ‘A probabilistic metric for the validation of computational models’

**DOI:** 10.1098/rsos.191986

**Published:** 2019-12-04

**Authors:** Ksenija Dvurecenska, Steve Graham, Edoardo Patelli, Eann A. Patterson

*R. Soc. open sci.* **5**, 180687. (Published 14 November 2018). (doi:10.1098/rsos.180687)

A small error has been highlighted by someone attempting to implement our validation metric described in this paper. The error occurs in equation (3.4), where $w_{k}$ should be $e_{k}$; and the same correction should be made in the expression in the text in the following line and in the text two lines above, which implies in the same line, that the word ‘weighted’ should be moved to appear before ‘sum’.

Hence, the second sentence on page 6 should read:

‘Once these three steps were completed, the errors, $e_{k}$_,_ were compared to the error threshold, $e_{th}$_,_ and the weighted sum of those errors less than the threshold computed to yield the validation metric, VM, i.e.

3.4 $$\mathrm{VM}=\underset{i}{\sum }w_{i}{∥}_{e_{k}<e_{th}}$$

where || is an indicator function which takes the value 1 when $e_{k}<e_{th}$ and otherwise has a value zero’.

The correction has no implications for the results or conclusion because the algorithm used to process the data, and provided in the Dryad Digital Repository, used the above version.

